# Behavior of an Inductive Loop Sensor in the Measurement of Partial Discharge Pulses with Variations in Its Separation from the Primary Conductor

**DOI:** 10.3390/s18072324

**Published:** 2018-07-18

**Authors:** Jorge Alfredo Ardila-Rey, Aldo Barrueto, Alvaro Zerene, Bruno Albuquerque de Castro, José Alfredo Covolan Ulson, Abdullahi Abubakar Mas’ud, Patricio Valdivia

**Affiliations:** 1 Department of Electrical Engineering, Universidad Técnica Federico Santa María, Av. Vicuña Mackenna 3939, 8940000 Santiago, Chile; aldo.barrueto@usm.cl (A.B.); alvaro.zerene@alumnos.usm.cl (A.Z.); patricio.valdivial@usm.cl (P.V.); 2 Department of Electrical Engineering, São Paulo State University, Av. Eng. Luiz Edmundo Carrijo Coube 14-01, 17033-360 Bauru, Brazil; bruno.castro@unesp.br (B.A.d.C.); alfredo.ulson@unesp.br (J.A.C.U.); 3 Department of Electrical and Electronics Engineering, Jubail Industrial College, P.O. Box 10099, 319261 Jubail, Saudi Arabia; abdullahi.masud@gmail.com

**Keywords:** partial discharges (PD), inductive loop sensor, primary conductor, frequency response, electrical insulation condition

## Abstract

Ideally, an insulation system must be capable of electrically insulating the active components of a machine or device subjected to high voltages. However, due to the presence of polluting agents or imperfections inside or on the surface of the insulation, small current pulses called partial discharges (PDs) are common, which partially short-circuit the insulation and cause it to lose its insulating properties, and thus its insulation capacity, over time. In some cases, measurements of this phenomenon are limited by the type of sensor used; if it is not adequate, it can distort the obtained results, which can lead to a misdiagnosis of the state of the device. The inductive loop sensor has experimentally been demonstrated to be capable of properly measuring different types of PDs. However, because of its current design, there are several practical limitations on its use in real devices or environments. An example is the presence of a primary conductor located at a fixed distance from the sensor, through which PD pulses must flow for the sensor to capture them. In this article, the sensor’s behavior is studied at different separation distances from the line through which the PD pulses flow. In addition, the measuring capacity of the sensor is tested by removing the presence of the primary conductor and placing the sensor directly over the line through which the PD pulses of a real device flow.

## 1. Introduction

Partial discharges (PDs) can be considered to be localized current pulses that partially short-circuit or bridge the insulation between two conductors and are the product of an electric field concentration in a specific region of the material [1,2]. In general, PDs are measured as low-magnitude and short-duration transient signals, whose spectral content can exceed 50 MHz depending on the type of PD source, the sensor used, the bandwidth of the acquisition system, the measuring circuit and the signal path [3]. As has been documented in several studies [4,5,6,7,8], PD activity in an insulation system can cause a progressive deterioration of the material due to the presence of physicochemical processes that are the product of ion/electron bombardment on the insulator walls. For this reason, early detection of this phenomenon is important to adequately diagnose the device and take immediate actions regarding its maintenance [9,10].

To accurately measure the PD activity in an electrical asset, it is recommended to use instruments capable of capturing and storing high-frequency electrical pulses on the order of MHz [3], obtain the largest amount of information possible about these signals, and then perform an adequate separation and identification process of the type or types of PD, which is important for the final diagnosis of the device or machine being tested [11,12,13,14].

Based on these factors, several types of sensors that can be coupled to standard measuring circuits have been experimentally and commercially developed. These sensors have proven to be effective in some environments, such as in laboratories or industries [3,15,16,17,18]. Examples include high-frequency current transformers (HFCTs), Rogowski coils (RCs) and inductive loop sensors (ILSs) [3,16,17,18,19,20,21,22]. These sensors generally consist of one or several copper turns that wrap a magnetic or non-magnetic core located around (HFTCs and RCs) or near (ILSs) the line through which the PD pulses propagate. In addition, they are practical for performing any measurement because they do not require galvanic contact with the measuring circuit.

The ILS is simple and inexpensive to implement because it basically consists of a copper turn printed on a circuit board, which must be placed parallel to the conductor through which the pulses to be measured flow. Therefore, measurements of the signals associated with PDs using this sensor result in very practical off-line measuring processes.

Several previous studies have demonstrated the versatility of this sensor for measurements in controlled environments [3,15,21,22,23,24]. However, in its current state, the physical configuration of the sensor when connected to the measuring circuit is very limited for some real applications because it is set at a fixed distance of 1 mm from the primary conductor through which the PD pulses flows. This limits the use of these sensors in on-line measuring systems with real test objects, such as generators, transformers and high-voltage cables, because there is no easy access to the connection points to perform the galvanic coupling with the primary conductor of the sensor. Thus, in many cases, the measurement must be performed directly on the conductors of the device, and there may be several insulation layers that prevent the sensor from being located close to the line through which the discharges flow. Furthermore, no study has described the sensor’s behavior during the measurement process when varying its separation from the primary conductor or alternatively by removing it during the measurement process.

Based on these factors, this article studies the sensor frequency response with increasing separation from the primary conductor theoretically, using the finite element method and experimentally. In addition, the variations in the electrical parameters that are part of the ILS are identified to more accurately determine the frequency response of the sensor for different separation distances. Finally, two experimental tests are accomplished with a real test object and a point–plane configuration using the ILS without a primary conductor.

This article is divided in five sections. Section 2 presents the description of the experimental measurement prototype. After that, in Section 3 the article presents the parameters of the ILS and then, in Section 4, the experimental measurements on the two test objects and the results are discussed. The article is finalized by the conclusion in Section 5.

## 2. Description of the Adjustable Experimental Measurement Prototype

**Figure 1:**
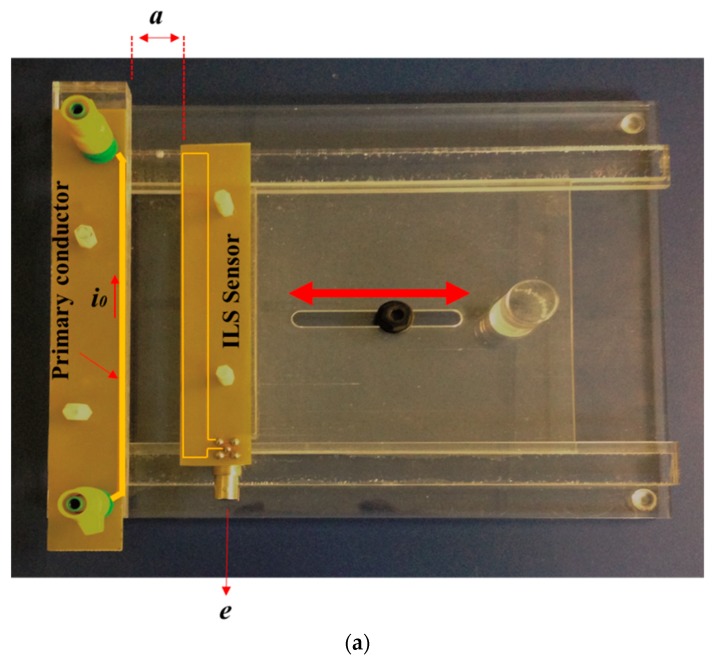

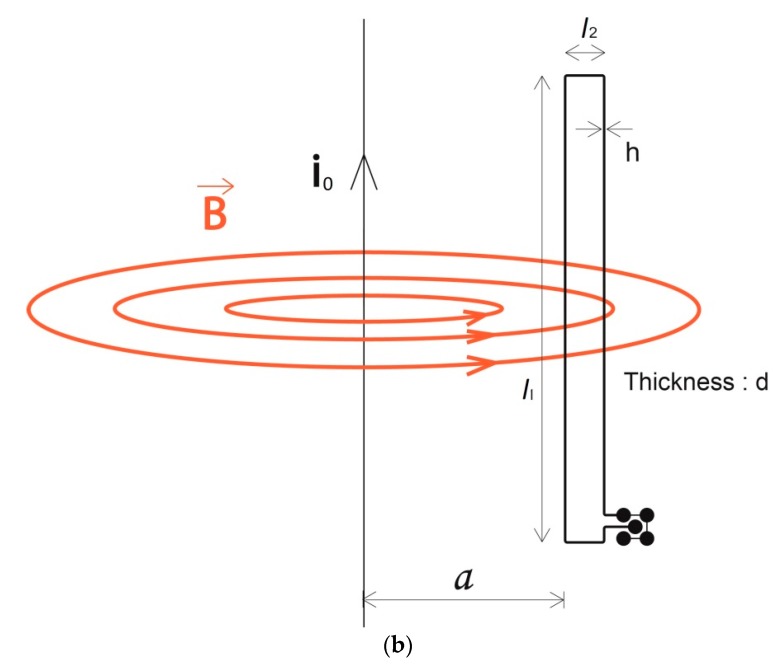
(**a**) Adjustable experimental measurement prototype; (**b**) scheme of the ILS used.

Figure 1a shows the experimental prototype used for the experimental characterization of the ILS, which can be manually adjusted to different separation distances (*a*) from the primary conductor (the line through which the PD pulses flow). As indicated in Section 1, the objective of varying *a* is to emulate the separation between the sensor and the line through which the PD pulses flow in a real device, since the line can contain insulation whose thickness can limit the close placement of the sensor.

In addition, the primary conductor in the experimental prototype was designed to easily connect its ends to any point on a measuring circuit. The idea of keeping the primary conductor in the proposed prototype is based on better controlling the distance between the sensor and the line through which the PD pulses flow. Thus, once the characterization of the sensor for different distances is performed, the primary conductor can be omitted, and the sensor can be placed in the same device that is being monitored without the need for a galvanic coupling.

The sensor incorporated into the experimental measurement prototype was similar to that used in [21] but does not use a fixed distance to the primary conductor.

In general, the sensor was based on a conducting rectangular loop printed on a circuit board. As with most of the inductive sensors used in PD measurements, the operating principles are governed by Ampere’s and Faraday’s laws [16,17,18], where the current pulses associated with the PD that flows through any conducting line generate a magnetic field that couples to the sensor and induces a voltage *e* in its terminals. Mathematically, the open-loop induced voltage can be represented according to Equation (1):

(1) $$e=M\frac{di_{0}}{dt}$$

where *i*_0_ is the current associated with the PD pulses, and *M* is the mutual inductance between the loop and the primary conductor.

In Figure 1b, *l*_1_ and *l*_2_ are the lengths of the rectangle that make up the sensor, *h* is the sensor’s width, *d* is its thickness, and *a* is the distance between the sensor and the primary conductor.

**Figure 2:**
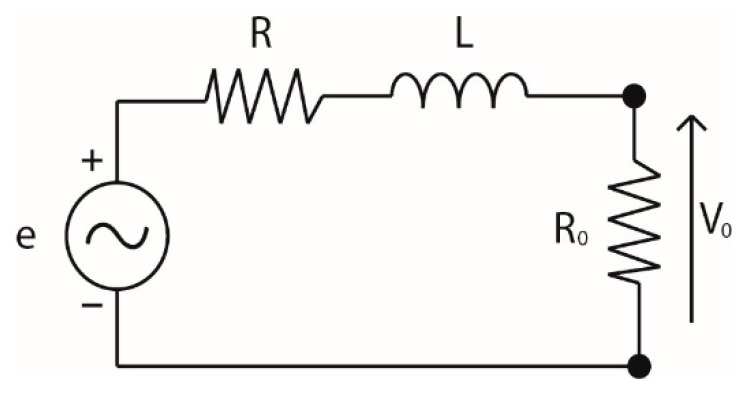
Equivalent circuit of the ILS.

To obtain the transfer function that describes the sensor’s behavior based on its electrical parameters, the lumped parameter model was applied as described in [15]; this was used to obtain the sensor’s equivalent circuit as shown in Figure 2, where *R* corresponds to the resistance of the inductive loop, *L* is the loop’s inductance, *V*_0_ is the voltage that occurs in the terminals of the sensor once it is connected to the measuring instrument, and *R*_0_ is the input impedance of the measuring system, which in this case is 50 Ω.

From this circuit, the transfer function shown in Equation (2) was obtained, where the loop’s resistance was neglected because it is much lower than the input impedance of the measuring system.

(2) $$\;\frac{V_{0}\left(s\right)}{I_{0}\left(s\right)}=\frac{sMR_{o}}{sL+\left(R+R_{0}\right)}\approx \frac{sMR_{o}}{sL+R_{0}}\;$$

It must be noted that any variation in the geometric parameters of the sensor—*l*_1_, *l*_2_, *h* and *d*—will generate changes in its electric parameters, considerably altering the bandwidth and sensitivity and in some cases will generate reflection problems [21,22].

## 3. Electrical Parameters of the ILS

In this section, the electrical parameters *R*, *L* and *M* that are part of the sensor’s transfer function are characterized by three methods: theoretically; using the finite element method; and experimentally. These analyses were performed to determine if the result obtained for each parameter is similar in each of the methods and to identify any variations in the electrical parameters with increasing or decreasing *a*.

The dimensions of the sensor were *l*_1_ = 120.4 mm, *l*_2_ = 10.4 mm, *h* = 0.35 mm, and *d* = 0.035 mm. The procedures described below are based on these dimensions.

### 3.1. Theoretical Calculation of the Electrical Parameters

A series of mathematical expressions from the technical literature were used for this procedure to obtain the theoretical values of *L* [25], *M* [21], and *R* [26] as shown in Equations (3)–(5):

(3) $$L=\frac{\mu_{0}}{\pi }\left[l_{1}ln\left(\frac{2l_{2}}{h}\right)+l_{2}ln\left(\frac{2l_{1}}{h}\right)\right],$$

(4) $$M=\frac{\mu_{0}}{2\pi }l_{1}ln\left(\frac{a+l_{2}}{a}\right),$$

(5) $$R=\frac{\rho lt}{hd\left(1-e^{-x}\right)}k_{c}$$

where *µ*_0_ is the magnetic permittivity of a vacuum, *l*_1_, *l*_2_, *h* and *d* are the parameters of the copper loop, ρ is the resistivity of the material (1.72 × 10^−8^ Ωm for copper), lt is the total length of the loop (*l*_1_ + *l*_2_), *k_c_* corresponds to the increase of the resistance due to current crowding, which can be expressed as $1+F_{\left(f\right)}\left(\frac{1.2}{e^{\frac{2.1d}{h}}}+\frac{1.2}{e^{\frac{2.1h}{d}}}\right)$, where *F_(f)_* describes the variation of the current crowding as a function of the frequency, which can be represented as $1-e^{-0.026p}$, $p=\frac{\sqrt{A}}{1.26\delta }$, *A* is the cross-sectional area of the loop, $\delta =\frac{66.6}{\sqrt{f}}$ is defined as the skin depth, and *x* is the effective thickness variation of the sensor due to the frequency and is defined as $x=\;\left(\frac{2\delta }{d}\left(1+\frac{d}{h}\right)+8\frac{{\left(\frac{\delta }{d}\right)}^{3}d}{h}\right)/\left({\left(\frac{h}{d}\right)}^{0.33}e^{-\frac{3.5d}{\delta }}+1\right)$.

According to Equation (3), *L* only depends on constant parameters; thus, the self-inductance can easily be obtained by replacing the sensor’s geometrical values in the equation. In this case, the result obtained is 223.9 nH.

**Figure 3:**
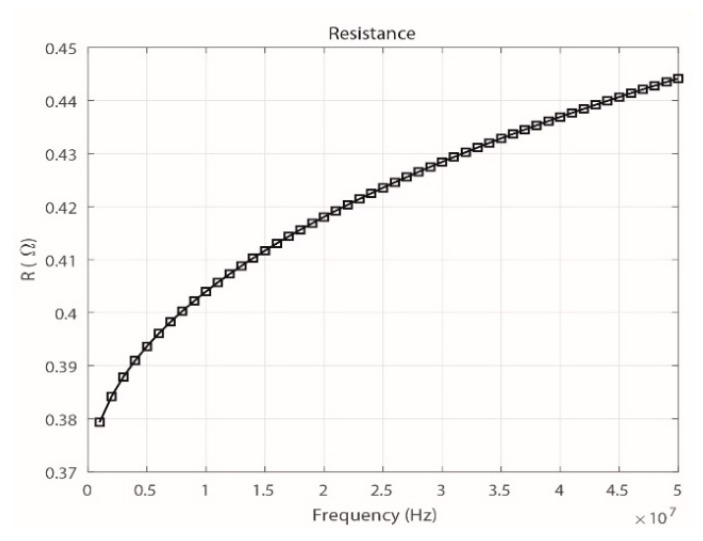
Theoretical behavior of *R* with increasing frequency of the signal.

According to Equations (4) and (5), the values of *M* and *R* depend on variable parameters such as the separation distance *a* and the signal frequency *f*. When calculating *R* for different frequency ranges between 0.1 MHz and 50 MHz (Figure 3), an increase of the resistance is observed. However, the value of *R* is so low compared to the input impedance of the measuring instrument (50 Ω), even for the maximum frequency analyzed of 50 MHz (*R* = 0.4442 Ω), that it is neglected.

**Figure 4:**
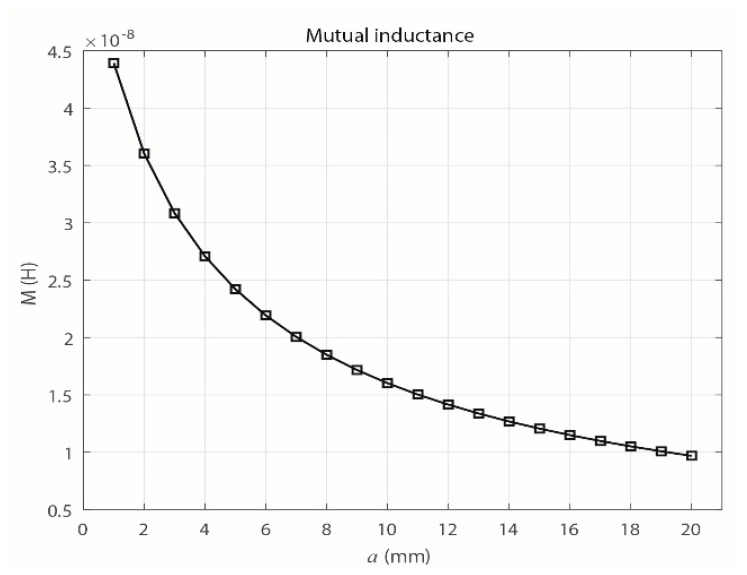
Theoretical behavior of the mutual inductance with increasing *a*.

On the other hand, as shown in Figure 4, the value of *M* decreases as *a* increases. This decrease of *M* is produced when the inductive loop is separated from the primary conductor, which causes the magnetic flux that links the circuits to decrease. As shown in Section 3.4, which describes the frequency response of the sensor, the maximum value of *a* is set at 20 mm because for higher values, the signal provided by the sensor has a very low magnitude, which makes measuring the signals difficult because the acquisition is limited by the minimum measurable magnitude stipulated by the technical characteristics of the measuring system.

### 3.2. Calculation of the Electrical Parameters Using the Finite Element Method

**Figure 5:**
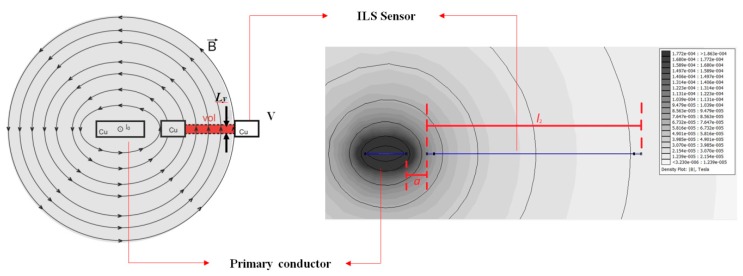
Representation of the distribution of *B* in FEMM.

The calculation of the electrical parameters from the finite element modelling was performed using the Finite Element Method Magnetics (FEMM) software. In this software, a magnetic model of the sensor was implemented through a two-dimensional configuration of each of the conducting sections, including the primary conductor (both on copper) (Figure 5).

Based on the results provided by the software, Figure 5 shows the distribution of the magnetic field generated by simulating a current *I* of 1 A through the primary conductor in the direction out of the page. A magnetic flux Φ that links the loop is obtained from the field distribution, and the flux is divided by the current to obtain the value of *M*.

In addition, FEMM provides the volume integral of *B* for an arbitrary region. In this case, a small region was chosen so the magnetic field lines would be as straight as possible in the region. The chosen volume is shown in Figure 5 as “*vol*”, whose cross-sectional area is $A=l_{y}(l_{2}-h)$, where $l_{y}=0.01\;\mathrm{mm}$, and the volume depth is $l_{1}-h$. Therefore, the value provided by FEMM can be described according to Equation (6):

(6) $$\int_{vol}Bdvol=\int_{l}\int_{S}BdS\;dl\;$$

where l corresponds to the vertical integration variable in the region *vol*, and *S* corresponds to the cross section crossed by the magnetic flux, which is the rectangular area of the loop, $S=(l_{1}-h)\times (l_{2}-h)$.

The magnetic field in the vertical coordinate is considered to be homogeneous because $l_{y}$ is very small; therefore, $\int_{vol}Bdvol=\int_{l}\int_{S}BdS\;dl=l_{y}\Phi$. Thus, the coefficient between the magnetic flux and the current I corresponds to the mutual inductance, as shown in Equation (7):

(7) $$M=\frac{\int_{vol}Bdvol}{l_{y}I}\;\;\;$$

**Figure 6:**
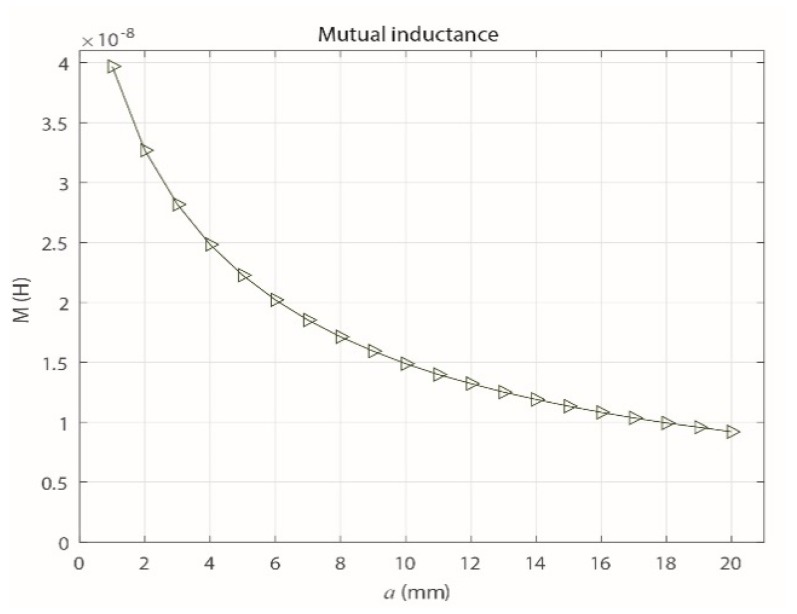
Mutual inductance obtained with FEMM with increasing *a*.

To vary the distance *a* in the finite element simulation, a routine was implemented with the Matlab software, which interacted with FEMM. This procedure was used to obtain the value of *M* for each of the distances. Figure 6 shows that with this method, the behavior of *M* is similar to that obtained theoretically (Figure 4), where *M* tends to decrease as *a* increases.

The geometrical configuration implemented in FEMM for the complete modelling of the loop was based on two cross sections of the loop, one for each pair of parallel sides. Each cross-sectional view was formed by the cross sections of the copper conductors and thus obtained the self-inductance of the shorter sides of the loop and the self-inductance of the longer sides. To calculate the self-inductance *L*, it was necessary to again simulate a current of 1 A but this time flowing through the inductive loop. This obtained the magnetic energy *W* due to the current flow; thus, the value of the self-inductance *L* is determined through Equation (8). The total inductance of the sensor is obtained from the sum of the self-inductances obtained for each pair of sides of the loop; *L* = 252.9 nH.

(8) $$\;L=\frac{2W}{I^{2}}=2W\;$$

To obtain the value of *R*, the losses *P* in the copper are obtained for each longitudinal line of the sensor with a current of 1 A as shown in Equation (9).

(9) $$\;R=\frac{P_{Copper}}{I^{2}}\;$$

Likewise, the value of *R* for the maximum frequency analyzed (50 MHz) is 0.45213 Ω. This value of *R* is very similar to that shown in Figure 3 for the same frequency band.

### 3.3. Experimental Calculation of the Electrical Parameters

Two devices were used for the experimental measurements of *L* and *R*: an RLC meter to obtain the value of the inductance and a Kelvin bridge, with which the value of the resistance was recorded by direct measurement on the inductive loop terminals. During the measurements, it was necessary to record the previous values of the cables with which the measurement was performed and then to subtract those values from the measurements performed on the inductive loop; therefore, very short cables were used.

The measurements performed with both instruments showed that the resistance *R* was 0.585 Ω and that the self-inductance *L* was 250.1 nH.

It should be noted that the value obtained for *R* with the Kelvin bridge is for a direct current (DC); therefore, this value tends to be different from the values obtained at high frequencies with the previous procedures (theoretically and FEMM). In addition, it was not possible to obtain the values of *R* as a function of the frequency due to the self-limitations of the laboratory in which the characterization of the sensor was performed; an impedance analyzer was not used to perform more accurate measurements. However, during the characterization of the sensor, experimental measurements of these parameters are not crucial because, as indicated in Section 3.1, the value of *R* is neglected from the transfer function of the sensor because its value is negligible compared with the input impedance of the measuring system. Therefore, the experimental measurement with the Kelvin bridge was only performed to verify that the value of *R* was of the same order of magnitude as those obtained theoretically and by FEMM.

**Figure 7:**
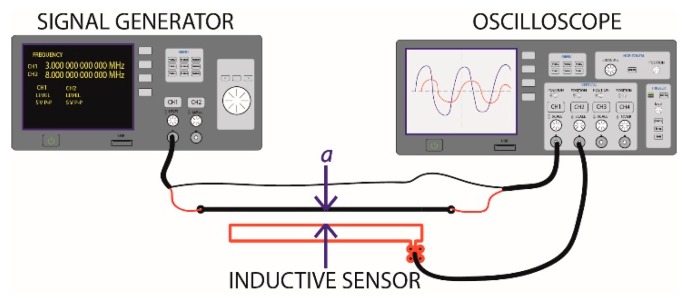
Experimental configuration for the characterization of *M* and the frequency response of the ILS.

The experimental configuration shown in Figure 7 was implemented for the measurement of *M*, in which a signal generator capable of generating sinusoidal signals of up to 60 MHz was connected to a commercial oscilloscope (channel 1) with a sampling frequency of 4 GSa/s and a bandwidth of 100 MHz. The input impedance of the oscilloscope (50 Ω) served as the charge for the signal generator, whereas the inductive loop was connected to channel 2 of the oscilloscope. A sinusoidal signal with amplitude of 5 *V_pp_* and a frequency of 1 MHz was configured in the generator, and the shapes of the generator wave *V_gen_* and the inductive sensor *V*_0_ were recorded by the oscilloscope to obtain the RMS values of both signals. The selection of the signal frequency was carried out randomly because, as indicated in Equation (4), *M* does not depend on the signal frequency, and the same values of *M* can be obtained for any frequency.

**Figure 8:**
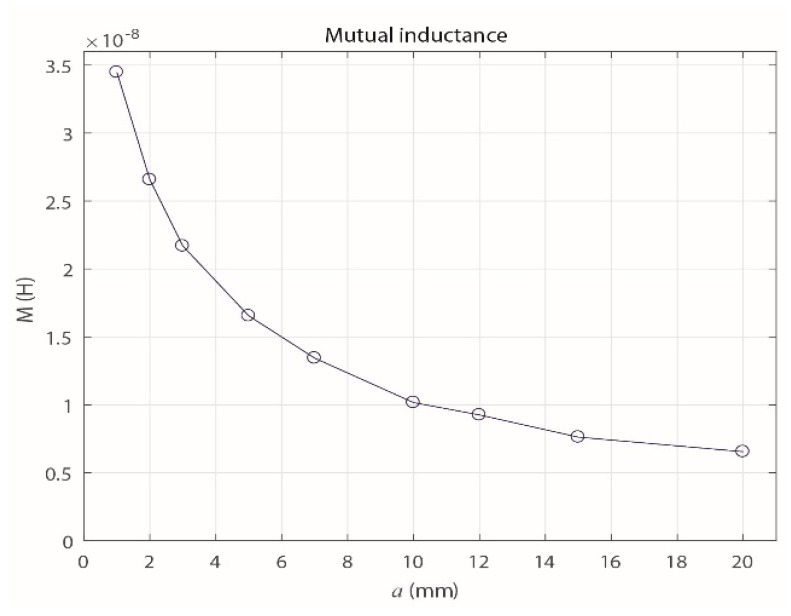
Experimental behavior of the mutual inductance with increasing *a*.

Equation (10) was obtained from Equation (2) and used to calculate the experimental mutual inductances of the sensor for different values of *a* (1, 2, 3, 5, 7, 10, 12, 15 and 20 mm). The results are shown in Figure 8, and they experimentally confirm that *M* decreases as *a* increases.

**Figure 9:**
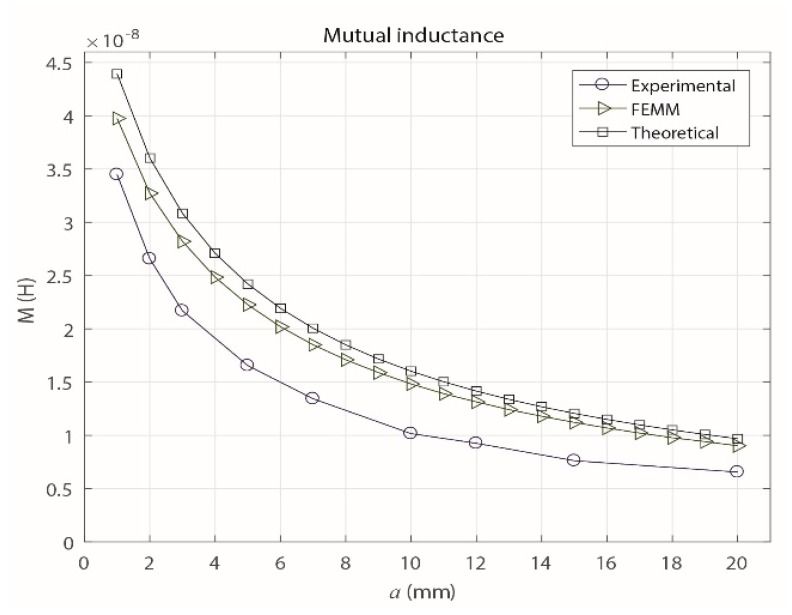
Comparison of the mutual inductances obtained with the three analysis methods.

Because the parameter *M* is most relevant when establishing the magnetic interaction of the sensor with the primary conductor as *a* varies, Figure 9 shows the results obtained for *M* with the three methods described in this section.

(10) $$M=\frac{\frac{V_{0}}{V_{gen}}\sqrt{{\left(\omega L\right)}^{2}+R_{0}^{2}}}{\omega }$$

Clearly, the methods gave similar results for the behavior of *M*, which decreases exponentially as *a* increases. However, due to the uncertainty in the measuring process and the possible errors in the manual adjustment of *a* in the experimental prototype, the experimental values of *M* vary slightly from the theoretical values and those from the FEMM simulation.

### 3.4. Characterization of the Frequency Response

In the previous section, the electrical parameters *R*, *L* and *M* of the inductive loop were validated, which confirmed that the experimental results obtained for these variables are consistent with those obtained theoretically and by the finite element simulation. Because these parameters are part of the transfer function of the sensor (Equation (2)), the frequency responses obtained from the sensor by the three procedures for each of the values of *a* are also expected to agree, which would validate the sensor’s behavior.

**Figure 10:**
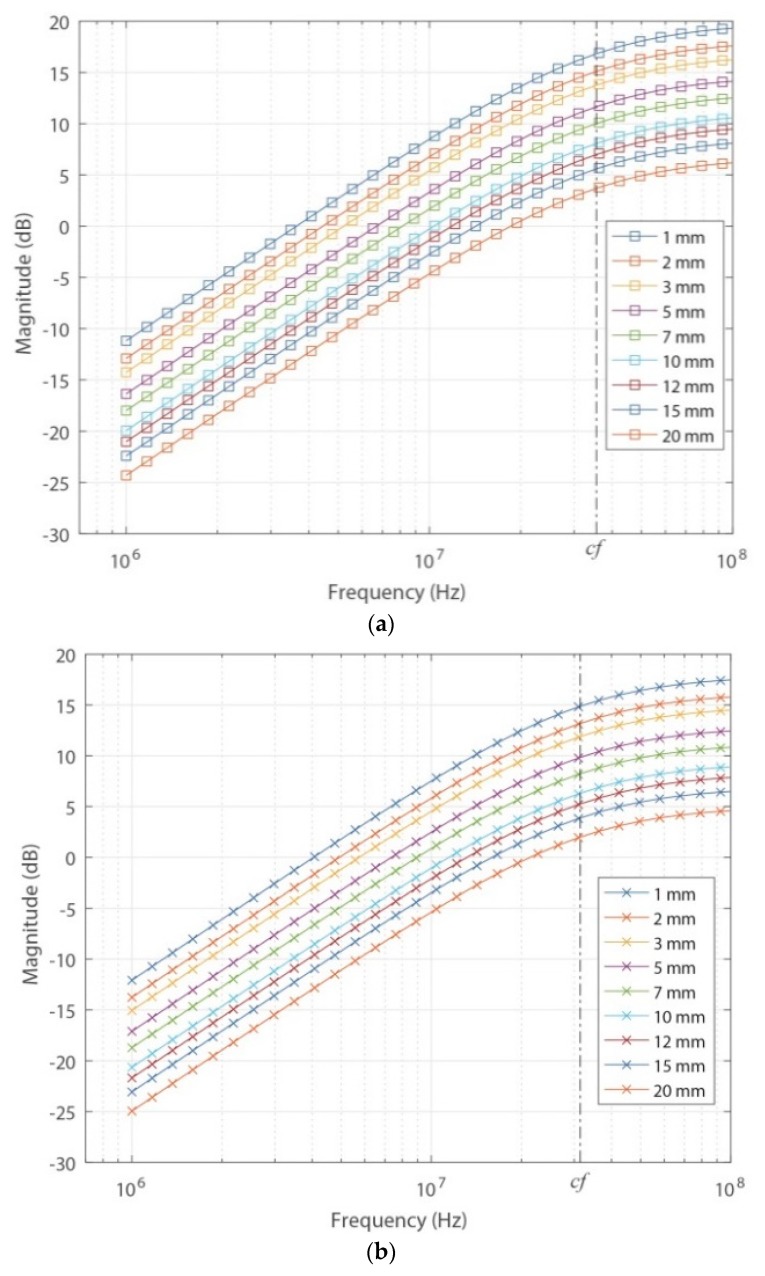
Frequency responses of the ILS for different values of *a*. (**a**) Theoretical; (**b**) by FEMM.

Figure 10a,b show the frequency responses of the sensor based on the theoretical parameters and from FEMM for different values of *a*. The values of *a* were the same as those used to obtain the experimental values of *M*, where the maximum tested value was 20 mm, because the sensor gain for higher separation values was very low and limited the measurement of low-magnitude PD pulses. To properly perform the measurements while maintaining higher levels of separation, it is necessary to increase the magnitude of the signal provided by the signal generator, which is unrealistic in these types of applications because PD pulses are generally of low magnitude.

Clearly, if this sensor is used in applications in which the transient signals to be measured have large magnitudes, higher values of *a* can be applied, and the pulses can be easily measured.

Figure 10a,b show that both methods have similar behaviors, where the sensor response is completely derivative up to the cut-off frequency (*cf*), which in this case was set at 35.54 MHz for the theoretical frequency response and at 31.30 MHZ for the frequency response by FEMM. This behavior of the sensor is consistent with that described in [3], where the sensor response for a fixed distance of *a* (1.016 mm) was also derivative up to the *cf* (34.69 MHz).

The results also confirm that as *a* increases, the sensor gain decreases proportionally to *M*, which is consistent because as was indicated in the previous section, the magnetic flux that links the sensor decreases as the separation between the sensor and the primary conductor increases. It is also important to note that no perturbation was observed in the sensor’s response, and no variation of the cut-off frequency occurred with increasing value of *a*.

Finally, the scheme in Figure 7 was used to obtain the experimental transfer function of the sensor for different frequencies. First, the distance *a* at which the response frequency of the sensor was measured was adjusted in the experimental prototype while simultaneously configuring a sinusoidal signal of 5 *V_pp_* in the generator for different frequencies, including 1, 3, 7, 10, 12, 15, 17, 20, 22, 25, 26, 28, 30, 32, 33, 34, 38, 40, 44 and 45 MHz. The shape of the wave provided by the generator and the inductive loop was recorded by the oscilloscope; the RMS values of both measurements and the current flowing through the primary conductor were obtained by $I_{0}=V_{gen}/R_{0}$. Finally, the sensor gain $G=20\log_{10}(V_{0}/I_{o})$ was calculated for each frequency, which allowed the transfer function to be obtained.

For frequencies above 45 MHz, an anomaly was observed in the experimental transfer function, which abruptly modified the frequency response of the ILS. Thus, the sensor’s behavior was only characterized up to this frequency. As indicated in [27], this behavior in some inductive sensors is associated with the effect of the connection cables on the experiment.

**Figure 11:**
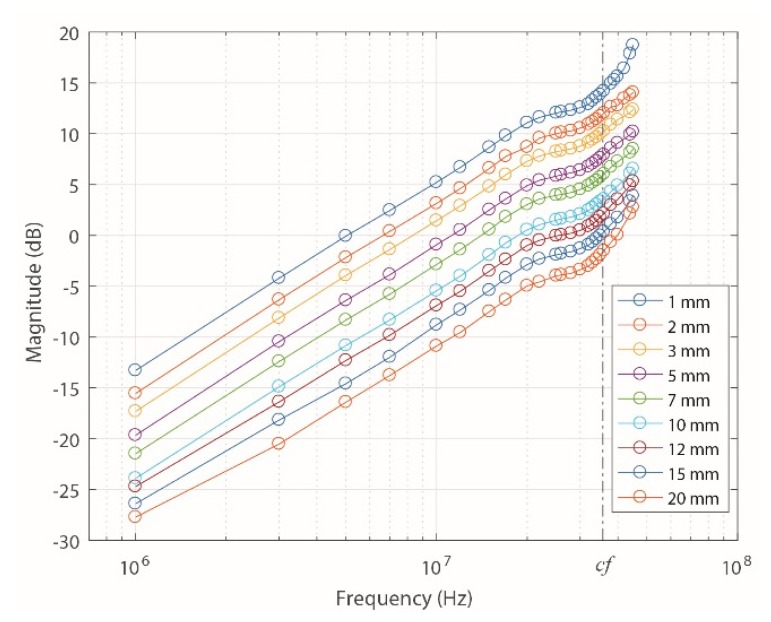
Experimental frequency responses of the ILS for different values of *a*.

The experimentally obtained results up to 45 MHz are shown in Figure 11.

As depicted in Figure 10a,b, the experimental frequency response of the sensor also has completely derivative behavior up to the *cf*, which is consistent with the cut-off frequencies obtained in the other two methods (Table 1). The experimental results also confirm that the increase of the distance *a* only decreases the magnitude of the frequency response due to the exponential decrease of the mutual inductance, which is interpreted in Equation (2) as a constant that multiplies the transfer function.

In addition, higher values of sensor gain were observed compared to those obtained theoretically and by FEMM, which is consistent with the results of *M* shown in Figure 9, where the experimental values of *M* were lower than the measured values of *a*.

## 4. Experimental Measurements

### 4.1. Power Transformer

In this experiment, the sensor’s behavior is compared for different values of *a* using a test object that is representative of the real environment. A measuring circuit that integrated an oil-paper-insulated distribution transformer (300 kVA, 12/0.42 kV) as the test object was used. The circuit is described in the IEC 60270 standard; it is composed of a coupling capacitor *C_k_* of 1nF connected in parallel with the test object, a measuring impedance *Z_n_* connected in series to *C_k_*, a high-voltage transformer as a high-voltage source, and a sensor element, which in this case was the sensor attached to the experimental prototype.

**Figure 12:**
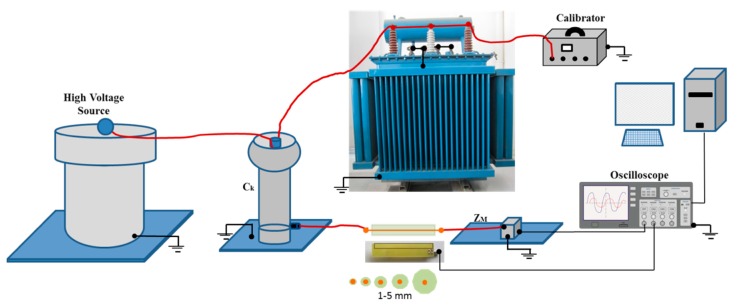
Experimental setup.

To generate a series of pulses with a stable magnitude and duration that would allow a more controlled evaluation of the sensor’s behavior, the high-voltage source was not powered; instead, a commercial calibrator commonly used in calibration processes for measuring PDs was used, which can inject charge pulses of up to 1000 pC. Figure 12 shows the experimental setup.

During the measurement process, the signals from the calibrator were injected into the measuring circuit through the test object by the high-voltage winding, and the low-voltage winding was short-circuited along with the transformer tank and connected to ground. Based on this experimental configuration, each of the pulses generated by the calibrator traveled along the measuring circuit from the test object to the coupling capacitor and then to the measuring impedance. Therefore, the ILS was coupled to the circuit between these two elements but without the primary conductor to be able to measure each of the signals in a less intrusive and more practical way.

This procedure was used to recreate a situation similar to that found in real measurement processes, in which it is not possible to easily perform a galvanic connection or cut to connect the primary conductor to the device being tested. The measurement can only be made if the sensor is placed parallel to the line through which the PD pulses flow, so the separation between the sensor and the line will only depend on the insulation’s thickness.

To establish different separations between the sensor and the line that connects *C_k_* and *Z_m_*, where the sensor was placed, five single-wire cable runs with different thicknesses of outer insulation (1, 2, 4, 10 and 20 mm) were used for the measurements. Because the primary conductor was absent, the ILS was placed tangentially to the outer surface of each cable run. Thus, in each case, the value of *a* corresponded to the cable thickness.

Each of the pulses that arrive at the sensor have very similar temporal and spectral behaviors to those of a PD because once they come out of the calibrator, they flow through the entire measuring circuit, including the test object. They tend to change their temporal and spectral behaviors due to the self-inductances and capacitances that they encounter throughout the circuit; therefore, the signal that arrives at the sensor is completely different from that produced by the calibrator. A similar process occurs in the insulation system of a real device because the signal produced by a disruption in a vacuole or defect is modified by the nature of the test object and the other components of the measuring circuit [28,29,30].

**Figure 13:**
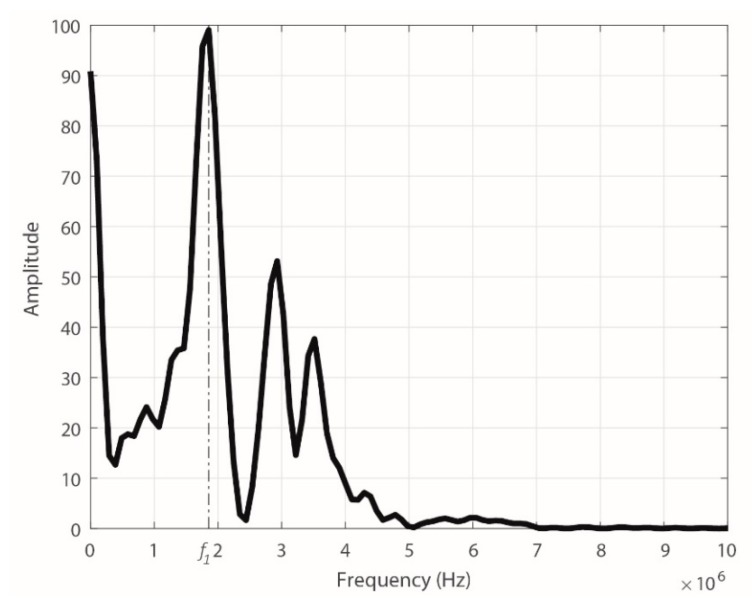
FFT of the signal captured by the ILS.

For the experimental setup and the subsequent measurement process, the calibrator was configured to repeatedly provide pulses of 500 pC. As example, Figure 13 shows the Fast Fourier Transform (FFT) of a pulse from the calibrator, which was captured with the ILS in the 1-mm-thick cable. The fundamental frequency (*f_f_*) of these types of pulses provided by the calibrator is 1.85 MHz.

**Figure 14:**
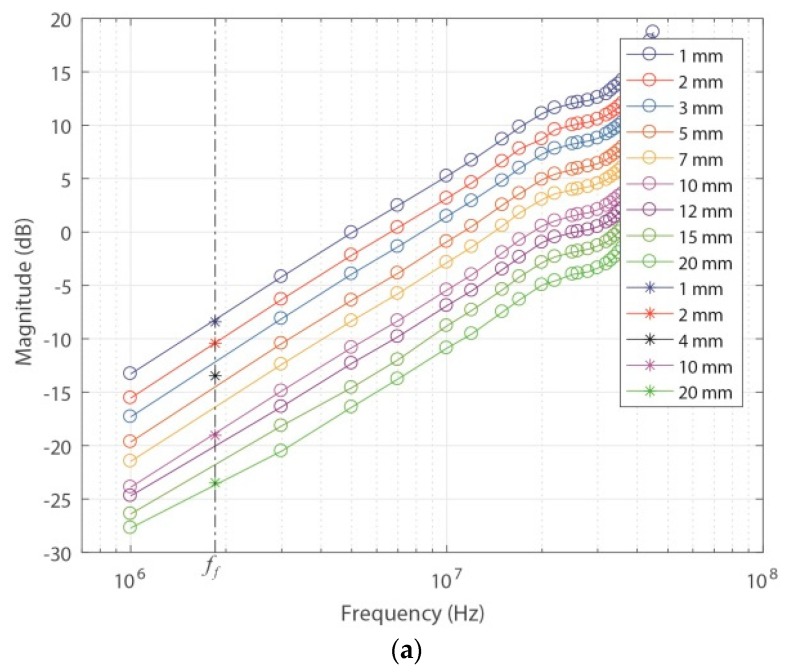

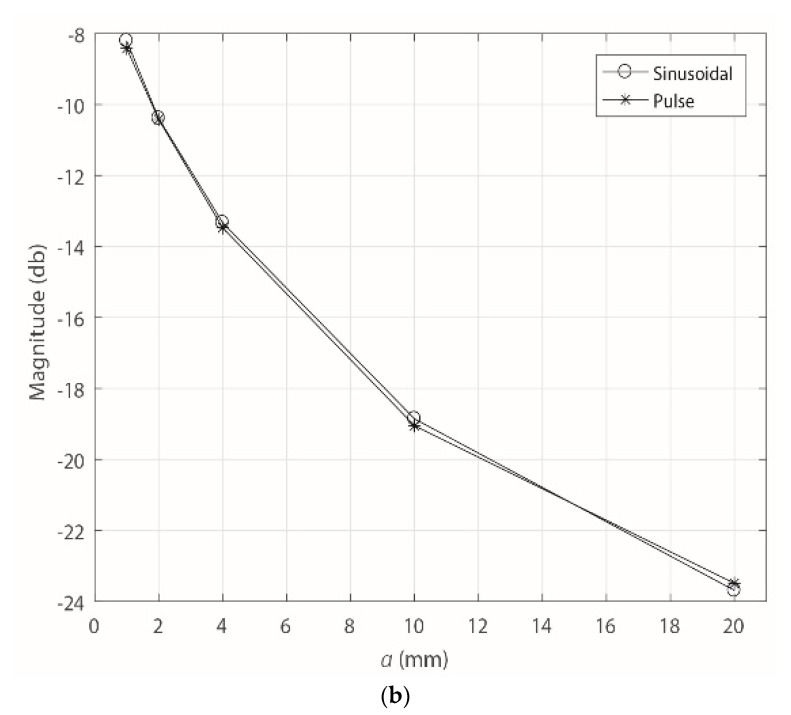
Comparison of the results obtained for 1.85 MHz. (**a**) Frequency response of the sensor; (**b**) sensor gain with a variation of the separation from the line through which the pulses flow.

Once the generation and acquisition in the experimental setup were controlled, the measurement process and the storage of the pulses that flowed through each of the cable runs (*V_gen_*) and those provided by the sensor (*V*_0_) in each case were performed. By applying the procedure used in Section 3.4, the gain value was obtained for the fundamental frequency of the pulses (1.85 MHz). These results were compared with those of Section 3.4 and are shown in Figure 14a.

In general, the sensor’s gain behavior over this frequency band for the cables with insulation 1, 2, 10 and 20 mm thick was consistent with the gain measured when the sensor was characterized with sinusoidal signals using the primary conductor in the experimental prototype and with the same values of *a*. Furthermore, for the case in which the cable run had 4-mm-thick insulation, the sensor’s response was as expected because an intermediate value to those obtained for the experimental curves of 3 and 5 mm, which were also obtained with the experimental prototype, was established.

Finally, Figure 14b shows the relation between the ILS gain and the increase of *a* for both experimental measurements. These results confirm that the characterization performed with the experimental prototype is consistent with the results obtained with the sensor using a real test object and disregarding the use of the primary conductor.

### 4.2. Point–Plane Experimental Test Object

The measurement circuit used for this experiment was similar to the one shown in Figure 12. As a test object a point–plane was used in order to generate corona PD pulses, which according to their nature have a low variability in terms of amplitude [11,12]. The calibrator was removed from the experimental setup since the PD pulses were generated from the energization of the test object. For the measurement of the PD pulses used as reference for the calculation of the sensor gain, a commercial HFCT with a bandwidth of 80 MHZ was coupled to the same ILS connection line through which the PD pulses flow. According to the configuration of the test object, the needle was placed above a metallic ground plane and the distance between the needle and the plane was adjusted to 12 mm. A stable PD activity was found for a voltage level of 4.8 kV.

**Figure 15:**
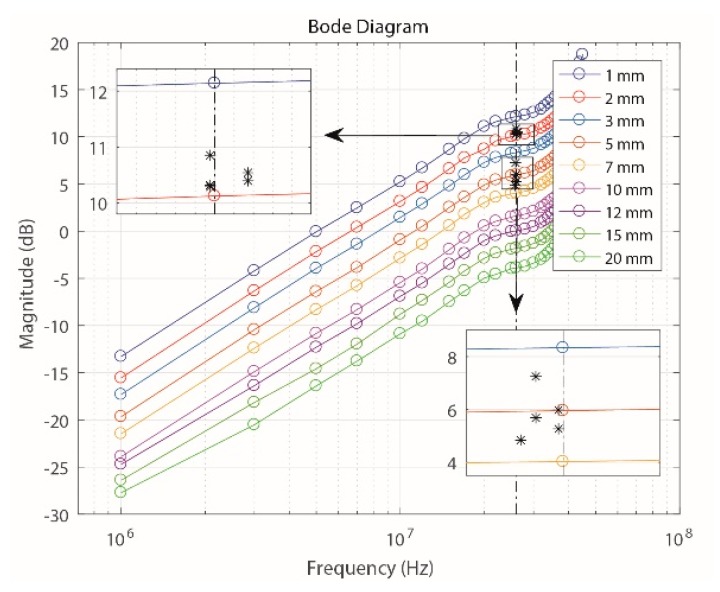
Comparison of the results obtained with the corona PD pulses for *a* values of 2 mm and 5 mm.

For this experiment, the measurement process was performed by placing the ILS on two cable runs with thicknesses of 2 mm and 5 mm. Although several hundred pulses were measured for each separation, only 5 pulses were used in each case for the calculation and the representation of the gain according to the sensor’s frequency response. The results are shown in Figure 15.

**Figure 16:**
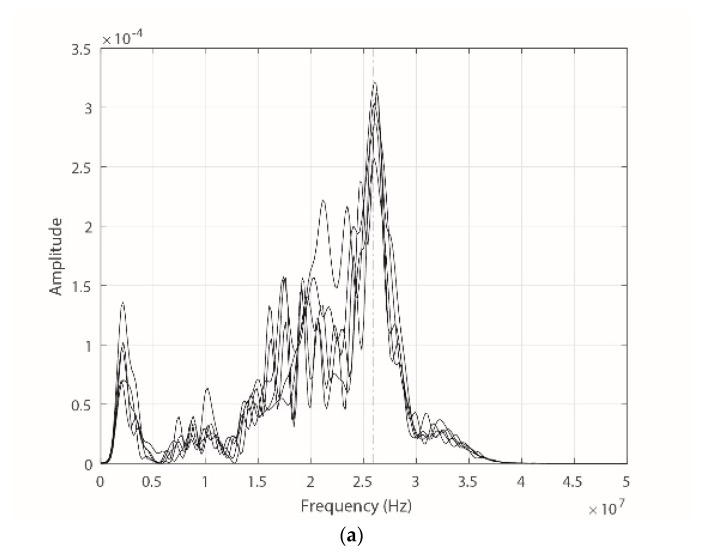

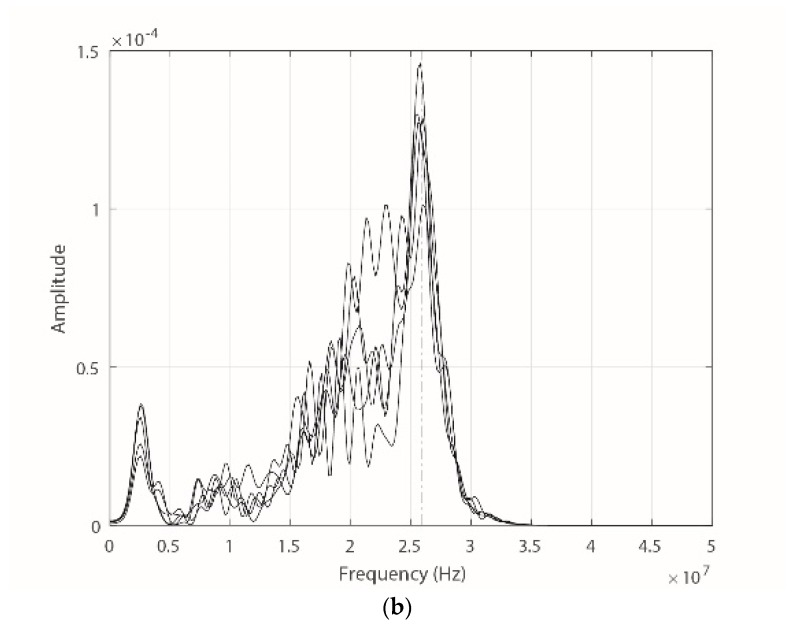
FFT of the signals captured by the ILS. (**a**) For 2 mm; and (**b**) for 5 mm.

As shown in Figure 15, for both separation distances, obtained from the fundamental frequency of the corona PD pulses (approximately 26 MHz in both measurements, see Figure 16), each group of pulses tends to take values of gain similar to those obtained when the sensor was characterized with sinusoidal signals in the experimental prototype. Although it is the same type of PD source, it is normal to find small variations in the fundamental frequency and the amplitude of the selected pulses for both values of *a*. These variations are also reflected in the two enlarged views on Figure 15. When changing the value of a, for this type of signals there are no variations also in the spectral power by frequency bands, which is concordant with the results obtained in Section 3.4 and in the previous section. Only important changes are observed in the magnitude of the spectral power, which decreases when increasing *a* from 2 mm to 5 mm. As described before, this behavior occurs due to the decrease in *M*.

## 5. Conclusions

This study characterized the behavior of an ILS with varying separation from the primary conductor through which pulses associated with the PD in the measurement process were circulated. The characterization was performed using three methods: theoretical; FEMM; and experimental. The three methods provided similar values for each of the electrical parameters that are part of the electrical model of the sensor. Some differences in magnitude were observed for *R* and *M* when they were obtained using the adjustable experimental measurement prototype. However, as described in Section 3.3, these variations are due to external factors, such as the lack of an impedance meter to obtain the value of *R* for different frequency ranges and errors in the manual adjustment of the separation distance in the experimental prototype, which could generate the variations observed in the experimental measurements of *M* for different values of *a*.

The frequency response of the sensor obtained with each of the methods with different values of *a* showed similar behavior in terms of the gain. Each method demonstrated that the sensor had a completely derivative behavior up to the cut-off frequency.

When the measurements were performed using a real test object attached to a normalized measuring system with the ILS without a primary conductor and measuring pulses of 500 pC, the frequency response of the sensor for the pulses’ fundamental frequency was very similar to the experimental response obtained directly with the experimental prototype and using sinusoidal signals provided by the commercial signal generator. When using the point–plane test object, the response of the sensor for the corona PD pulses in both measurements was also similar to that obtained with the experimental prototype, maintaining the same values of *a* (2 mm and 5 mm) and using the signal generator. These results confirm that the experimental prototype is useful for characterizing the sensor’s behavior for different separation distances from the line through which the pulses flow. The results also confirm that the sensor can be used directly on the measuring line of the device being tested without the need for a primary conductor, for which it would be necessary to modify a part of the measuring circuit to perform the measurement.

## Figures and Tables

**Table 1:** Cut-off frequencies in the ILS for each of the analysis methods applied.

Method	*F_c_* = *R*_0_/2π*L* (MHz)
Theoretical	35.54
FEMM	31.46
Experimental	31.83
